# Interleukin 26 attenuates osteoblast differentiation in osteoarthritis patients by activating COX2 and NF-κB pathways

**DOI:** 10.7150/ijms.102967

**Published:** 2025-02-26

**Authors:** Yi-Hsuan Lin, Yi-Hsun Wang, Yi-Jen Peng, Feng-Cheng Liu, Huey-Kang Sytwu, Chia-Pi Cheng

**Affiliations:** 1 Department and Graduate institute of Biology and Anatomy, National Defense Medical Center, Taipei, Taiwan.; 2 Graduate Institute of Life Sciences, National Defense Medical Center, Taipei, Taiwan.; 3 Department of Pathology, Tri-Service General Hospital, National Defense Medical Center, Taipei, Taiwan.; 4 Rheumatology/Immunology and Allergy, Department of Medicine, Tri-Service General Hospital, National Defense Medical Center, Taipei, Taiwan.; 5 National Institute of Infectious Diseases and Vaccinology, National Health Research Institutes, Zhunan, Miaoli County, Taiwan; Department of Microbiology and Immunology, National Defense Medical Center, Taipei, Taiwan.

**Keywords:** osteoarthritis, interleukin 26, osteoblast differentiation

## Abstract

**Aims:** Osteoarthritis (OA) represents the prevailing form of degenerative joint pathology. Recent investigations have revealed a heightened expression of interleukin 26 (IL-26) in various inflammatory arthritic conditions, including OA. However, the specific impacts and functions of IL-26 on osteoblasts (OBs) within the context of OA remain inadequately elucidated. This study aims to clarify the effects and underlying mechanisms of IL-26 by examining its influence on osteoblasts isolated from OA patients and a murine osteoblast cell line.

**Methods:** Human primary osteoblasts and mouse pre-osteoblast cells were subjected to treatment with β-glycerophosphate or concurrent treatment with IL-26 to observe the effects on osteoblast differentiation. The differentiation of osteoblasts was assessed through the expression of relevant genes using reverse transcription-polymerase chain reaction (RT-PCR). Key molecular mechanisms of downstream signaling pathways were examined through immunoblotting assays.

**Results:** Our results reveal that IL-26 mitigates osteoblast differentiation and reduces the expression of the marker alkaline phosphatase. Furthermore, the NF-κB downstream OB proliferated marker iNOS and inhibition OB differentiated marker LCN2 messenger RNA are up-regulated in IL-26 treated group. Also, phosphorylation and nuclear translocation of NF-κB p65 occur following IL-26 stimulation. Additionally, IL-26 enhances the downstream transcription factor cyclooxygenase-2 (COX2), a major player associated with iNOS. STAT1, the canonical receptor signaling pathway of IL-26 is activated.

**Conclusion:** In summary, our findings substantiate the role of IL-26 in osteoarthritis and identify it as a potential therapeutic target for intervention in osteoarthritic pathology.

## Introduction

Osteoarthritis (OA) represents a prevalent and debilitating joint disorder characterized by progressive articular cartilage degeneration, subchondral bone remodeling, and synovial inflammation 1. The pathogenesis of osteoarthritis (OA) includes a complex network of pro-inflammatory mediators and cytokines in the synovial fluid that exacerbate joint damage and promote degeneration through immune and inflammatory pathways 2.

Bone degeneration and destruction in osteoarthritis (OA) result from impaired bone-forming cells and overactive bone-absorbing cells. Several growth factors, such as bone morphogenic protein (BMP), transforming growth factor β (TGFβ), and parathyroid hormone (PTH), play a crucial role in promoting the development of stem cells into precursor osteoblasts 3. Previous studies have reported that growth factors and Wingless (Wnt) signaling pathways are crucial for osteoblast differentiation and function 4, 5. In addition to producing RANKL and MCSF to promote osteoclast differentiation, osteoblasts also produce osteoprotegerin (OPG), a negative regulator of osteoclast differentiation 6. OPG influences osteoclast destruction through the mutual regulation of RANK/RANKL/OPG, mediating osteogenesis, osteoclast maturation, and bone resorption 7, 8. Furthermore, osteoblasts have be proven to possess the properties of inflammatory cells, attracting white blood cells by producing IL-8 or CCL2, and thereby generating a more serious local inflammatory response 9.

Interleukin-26 (IL-26) is a member of the interleukin-10 cytokine family, playing a crucial role in immune response modulation 10. Although mice lack the IL-26 protein, previous research indicates that those possessing the IL-26-related receptors, IL-20RA and IL-10RB, show development comparable to that of the human system 11, 12. While the exact functions of IL-26 are still under investigation, emerging research suggests its involvement in the defense against microbial infections and the regulation of inflammatory cascades 13. A recent study revealed that IL-26 is broadly expressed in the serum and synovial fluid of patients with multiple types of arthritis, including OA 14. In the context of bone remodeling, previous studies found that IL-26 suppresses macrophage from osteoclastogenesis and promotes macrophages toward the M1 proinflammatory phenotype 15, 16. However, the direct role of IL-26 in bone formation in OA is still unknown. Therefore, our purpose is to clarify the functions of IL-26 cytokine in osteoblast differentiation to better understand the pathogenesis of OA.

## Materials and methods

### Cell line and reagents

The murine osteoblast cell line 7F2 was purchased from the Food Industry Research and Development Institute (FIRDI, Taiwan) and maintained as company description. Human IL-26 recombinant protein was purchased from MyBiosource (San Diego, USA). α-Tubulin, cyclooxygenase2 (COX-2), TATA-binding protein (TBP), Glyceraldehyde-3-phosphate dehydrogenase (GAPDH), Phosphorylated and non-phosphorylated STAT1, STAT3, and NF-κB p65 were obtained from Cell Signaling Technology (USA). The IL-6 ELISA kit was purchased from BioLegend (San Diego, USA). All other reagents were purchased from Sigma Chemical Co. (USA).

### Isolation and culture of primary human pre-osteoblast cell (HOB)

Bone sample specimens were obtained from patients with OA who underwent total knee replacement (TKR) in our institution (N = 20, Male: 6, Female: 14, mean age, 74.1 years; range 61-91 year). Specimens were minced into small pieces then incubated in antimicrobial solution containing 500 IU/mL of penicillin, 500 mg/mL of streptomycin, and 2.5 mg/mL of fungizone (Sigma-Aldrich) for 2-4 hours at room temperature, then washed with PBS vigorously to remove fat composition. Following the bone fragments were maintained in McCoy's 5A medium (Gibco, USA) supplemented with 10% FBS and 1x P/S (penicillin and streptomycin) at 37 °C in a humidified atmosphere of 5% CO_2_ for 2 weeks until the primary pre-osteoblast released from the bone fragment. The culture mediums were replaced every 2-3 days.

### Osteoblast differentiation

For osteoblast differentiation, 7F2 cells were seeded at 1x10^4^ cells per well, and HOB cells were seeded at 1x10^5^ cells per well in a 24-well plate. They were then stimulated with differentiation medium containing 5 mM β-glycerophosphate plus 50 μg/mL ascorbic acid (Sigma-Aldrich) concurrent with or without IL-26 (60 ng/ml) for 7 or 14 days 17. The culture media were replaced every 2-3 days. Experimental groups were divided into four; N: untreated normal 7F2 or HOB cells; IL-26: cells treated with 60ng/ml IL-26; bGP: cells treated with differentiation medium as a positive control for differentiation; and bGP+ IL-26: cells treated with both differentiation medium and IL-26.

### Real-time quantitative PCR

Total RNA from 7F2 and HOB cells was extracted using the NucleoSpin RNA Kit (Macherey-Nagel, Germany), following the manufacturer's instructions. One microgram of the purified RNA was converted to cDNA with the SensiFAST™ cDNA Synthesis Kit (Bioline, Meridian Bioscience). Gene expression markers were analyzed on the LightCycler 480 II platform, in a 20 µl reaction volume, using a 96-well white plate sealed with adhesive film (Roche, Germany). The resulting data were processed with LightCycler 480 software using the Relative Quantification mode, where values were normalized to GAPDH and calibrated against N groups. GAPDH was chosen as the internal control because it was found to be stable under our experimental conditions. PCR product specificity was verified by melt curve analysis, and gene expression was quantified relative to GAPDH using the 2^-ΔΔCt method. The thermal cycling protocol included an initial 15-minute step at 95 °C, followed by 40 cycles of 30 seconds at 95 °C, 30 seconds at 60 °C, and 1 minute at 72 °C, with a final 10-minute extension at 72 °C. The PCR primer sequences are provided in Table 1.

### Immunoblotting analysis

The cell cytoplasmic and nuclear extracts were separated according to the study by Peng *et al.* 15. Total protein was lysed by RIPA lysis buffer (GE healthcare), vortex and incubated on ice for 10 minutes subsequently centrifuged for 20 minutes at 14,000 rpm at 4°C. The equal amounts of protein were loaded into SDS-PAGE and immunoblotted by using specific antibodies for COX-2 NF-κB p65, phospho-NF-κB p65, STAT1, phospho-STAT1, STAT3, phospho-STAT3, α-tubulin, TBP, and GAPDH. All antibody dilution ratios were prepared according to the manufacturer's instructions, at 1:1000 for Western blotting. Phosphorylated proteins were probed first, then stripped and reprobed with the non-phosphorylated form.

### Alkaline phosphatase (ALP) and Alizarin red S staining (ARS)

After differentiation, 7F2 and HOB cells were fixed with fixation buffer (65:25:8 acetone/ citrate solution/10% formalin) for 15 min then staining with ALP staining kit (Sigma-Aldrich 86R-1KT) or 2% Alizarin red S (Sigma-Aldrich) according to the manufacture. After staining, the cells were washed with water and imaged by microscope.

### MTT assay

The murine and human cells (5×10^3^/well) were seeded in a 96-well plate with medium supplemented with 10% FBS and treated with various concentrations of IL-26 or IgG. IgG was used as a non-stimulating protein control in equal amounts. After 24 hours, the cells were treated with 0.5 mg/ml of 3-(4,5-dimethylthiazol-2-yl)-2,5-diphenyl- tetrazolium bromide (MTT) for 2 hours at 37℃. Cells were solubilized in 100 µl of DMSO then determined at 570 nm in an ELISA plate reader.

### Hematoxylin and eosin (H&E) staining and Immunofluorescence Staining

The morphology of bone specimens was examined using H&E-stained paraffin sections. For immunofluorescence staining, paraffin sections on coverslips were preserved in 10% formalin. After blocking nonspecific binding with 1% BSA in 0.1% Triton X-100, the sections were stained using a 1: 200 dilution of IL-26 (ab224198) primary antibody and a 1:200 dilution of goat anti-rabbit Alexa Fluor® 488 (011-540-003) secondary antibody (Jackson ImmunoResearch Laboratories Inc.). DAPI is used as a nuclear stain to visualize and quantify the location of cell nuclei. Image was performed with an Olympus SPOT Imaging System.

### Statistical analysis

Data were shown as means ± standard deviation and analyzed by one-way ANOVA and Newman-Keuls multiple comparisons in posttest with Prism software. p<0.05 was considered as statistically significant.

## Results

### The expression of IL-26 in the joints of osteoarthritis patients

**Figure 1:**
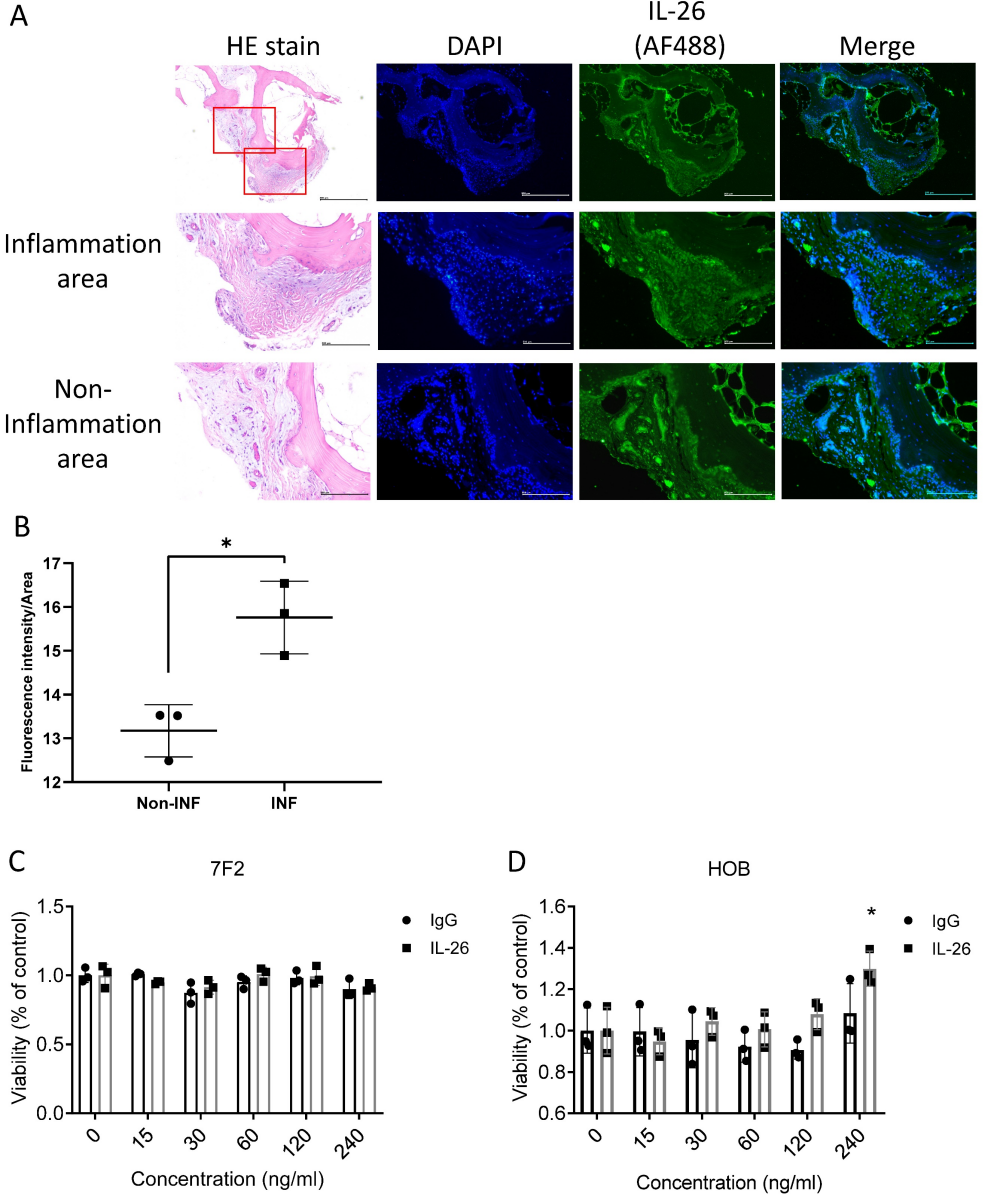
** IL-26 levels in osteoarthritis patients and impact on osteoblast cell viability.** (A) Bone sample specimens were obtained from patients with osteoarthritis (OA). A Histological examination was performed by HE stains. IHC staining was used to detect the expression of IL-26 **(Green)** in bone tissue. DAPI **(Blue)** was used for nuclear staining. (B) The immunofluorescent intensity on the bone surface per unit area was quantified using ImageJ software. (C) Mouse 7F2 pre-osteoblasts, (D) HOB was planted in 96-well plates and cultured with the specified concentrations of IL-26 or IgG control group for 24 hours. Add MTT (500 μg/ml) solution and incubate for 1 hour and measure the absorbance at 570 nm. All experiments were repeated at least three times, and the data represent the mean ± standard deviation. HOB: human osteoblast cells. *P<0.05 compared to the dose 0 ng/ml group.

To determine the location where IL-26 would be expressed in the joint of osteoarthritis. Bone samples sliced were observed by immunofluorescence staining of IL-26. The results showed that IL-26 was expressed on the bone surface and in bone inner cells in the inflammatory area more than in the non-inflammatory area, suggesting involvement with osteoblast and osteocyte cells (Fig. 1A, 1B). Thus, we used a murine osteoblast cell line, 7F2, and isolated human osteoblast cells (HOB), then treated the cells with different concentrations of IL-26 for 24 hours to measure cell viability by MTT. Our results showed that there was no effect on the viability of 7F2 and HOB cells when the IL-26 concentration was below 120 ng/ml (Fig. 1C, 1D).

### Effects of IL-26 on osteoblast differentiation and bone mineralization

**Figure 2:**
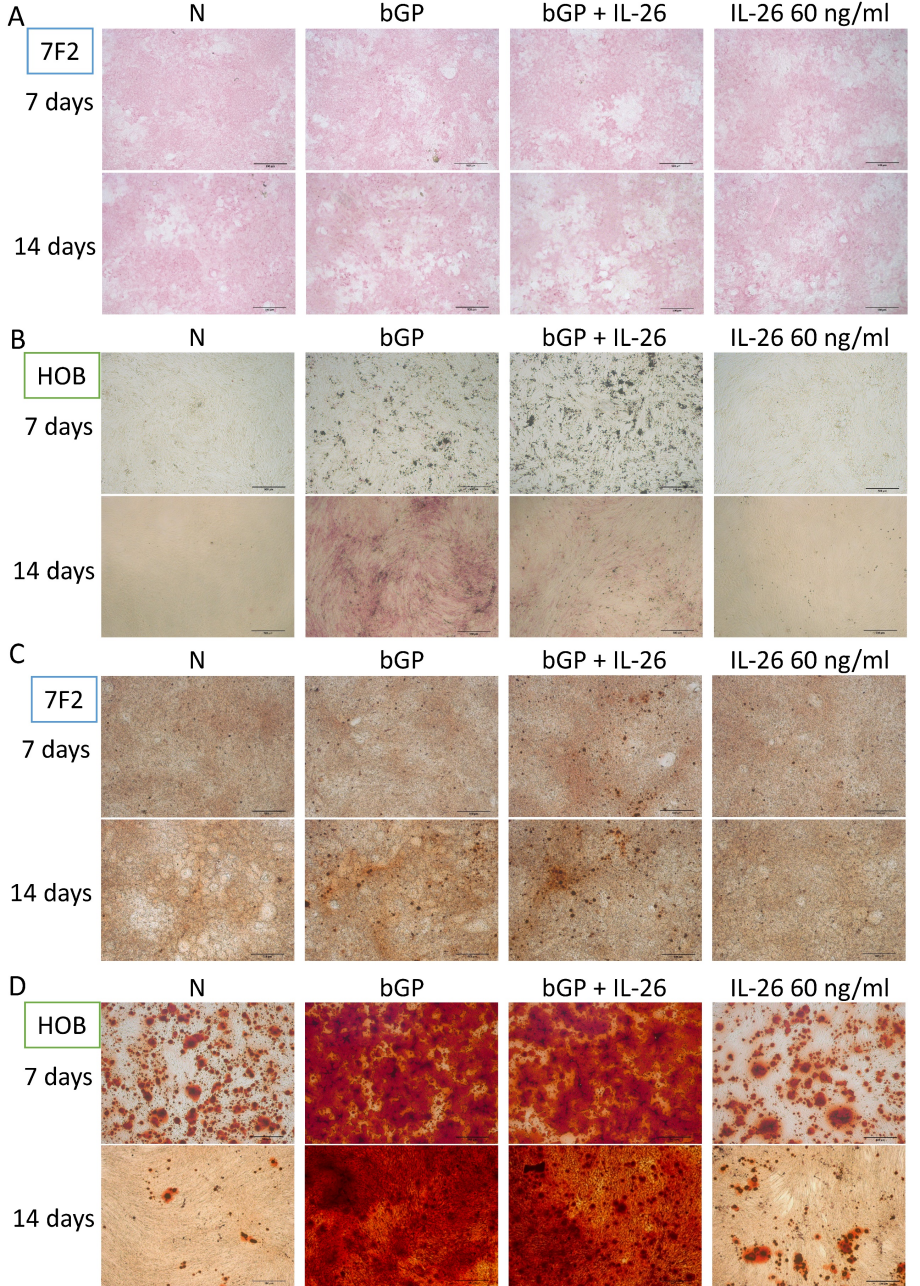
** Effect of IL-26 on osteoblast differentiation.** (A, C) 7F2 pre-osteoblasts and (B, D) HOB were differentiated using osteoblast differentiation medium (50 μg/ml ascorbic acid and 5 mM bGP) in the presence or absence of IL-26 60 ng/ml. (A, B) Alkaline phosphatase ALP staining and (C, D) alizarin red staining ARS were performed at 7, 14 or 21 days after differentiation medium stimulation. (bGP: β-glycerophosphate; bGP+IL-26: β-glycerophosphate+IL-26) ALP: Alkaline phosphatase assay.

The OA in bone degeneration and destruction may result from an impairment of bone-forming cells and an overactivity of bone-absorbing cells. Based on previous research, it is known that IL-26 suppresses bone-absorbing osteoclast (OC) formation 15. Here, we aim to clarify the direct effect of IL-26 on the bone-forming osteoblasts. We used the osteoblast differentiation model of 7F2 and HOB to test IL-26 function alone or in combination with osteoblast differentiation medium. Alkaline phosphatase (ALP), a marker of osteoblast differentiation, was stained on days 7 and 14 to analyze ALP activity 18. Our results showed that IL-26, when concurrently treated with osteoblast differentiation medium, inhibited ALP activity, but it had no effect when treated alone (Fig. 2A, 2B). Another marker of bone function, bone mineralization, was also used to assess osteoblast differentiation 17. After differentiation, IL-26 showed no effect on mineralization in the combined group (Fig. 2C, 2D).

### Effects of IL-26 on gene expression in osteoblast differentiation

**Figure 3:**
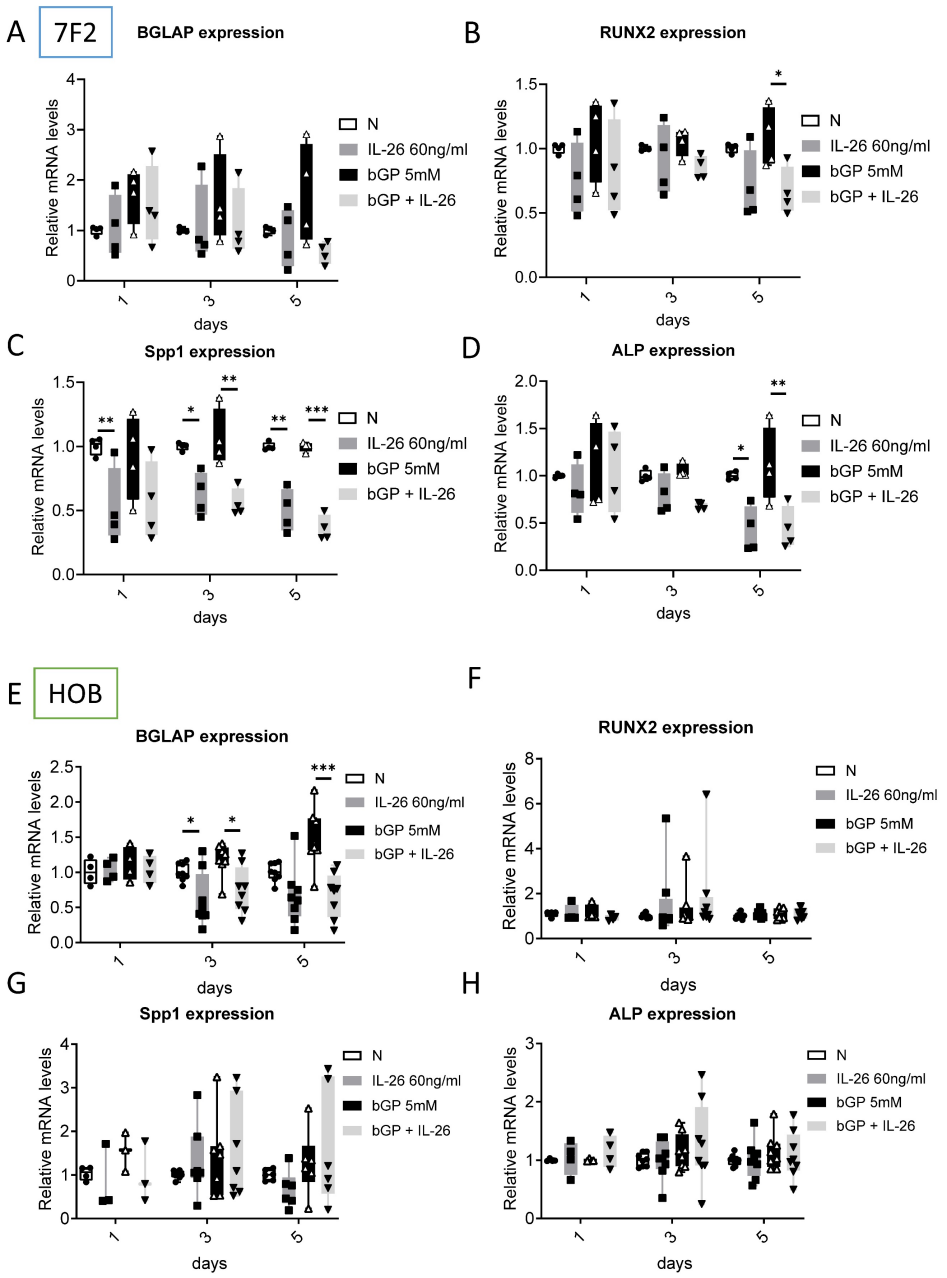
** Effect of IL-26 on mRNA expression during 7F2 and HOB osteoblast differentiation.** (A-D) 7F2 pre-osteoblasts and (E-H) HOB were differentiated using osteoblast differentiation medium (50 μg/ml ascorbic acid and 5 mM bGP) in the presence or absence of IL-26 60 ng/ml. Collect RNA on the 1st, 3rd or 5th day after differentiation stimulation, extract all the RNA and take 1 μg to transcribe into cDNA, and use the cDNA as a template to add mouse and human specific primers for real-time quantitative polymerase chain reaction, detection osteoblast differentiation gene markers BGLAP, RUNX2, Spp1, and ALP. The experiment was repeated at least three times, and the data represent the mean ± standard deviation. (*P<0.05, **P<0.01, ***P<0.001).

To further examine the suppressive effect of IL-26 on osteoblast differentiation, we evaluated the mRNA levels of related osteoblast markers after IL-26 treatment. We analyzed the expression of osteogenic marker genes, including bone gamma-carboxyglutamic acid-containing protein (BGLAP/Osteocalcin), which regulates bone turnover and bone mineralization, and runt-related transcription factor 2 (RUNX2), an important transcription factor involved in osteoblast differentiation during bone formation 19. Additionally, osteopontin (Spp1) plays a crucial role in bone formation and resorption 20, while bone sialoprotein (BSP) is the main non-collagen extracellular protein in bone components 21. Our results showed that in murine 7F2 osteoblasts, Spp1(63%), RUNX2 (38%), and ALP (60%) were significantly inhibited by IL-26 on day 5 (Fig. 3B-D). In HOB-differentiated cells, IL-26 downregulated BGLAP expression by approximately 50% only at the late stage (days 3 and 5) (Fig. 3E), with no significant changes observed in RUNX2, Spp1, and ALP (Fig. 3F-H).

### Effects of IL-26 on bone remodeling communication in osteoblast differentiation

**Figure 4:**
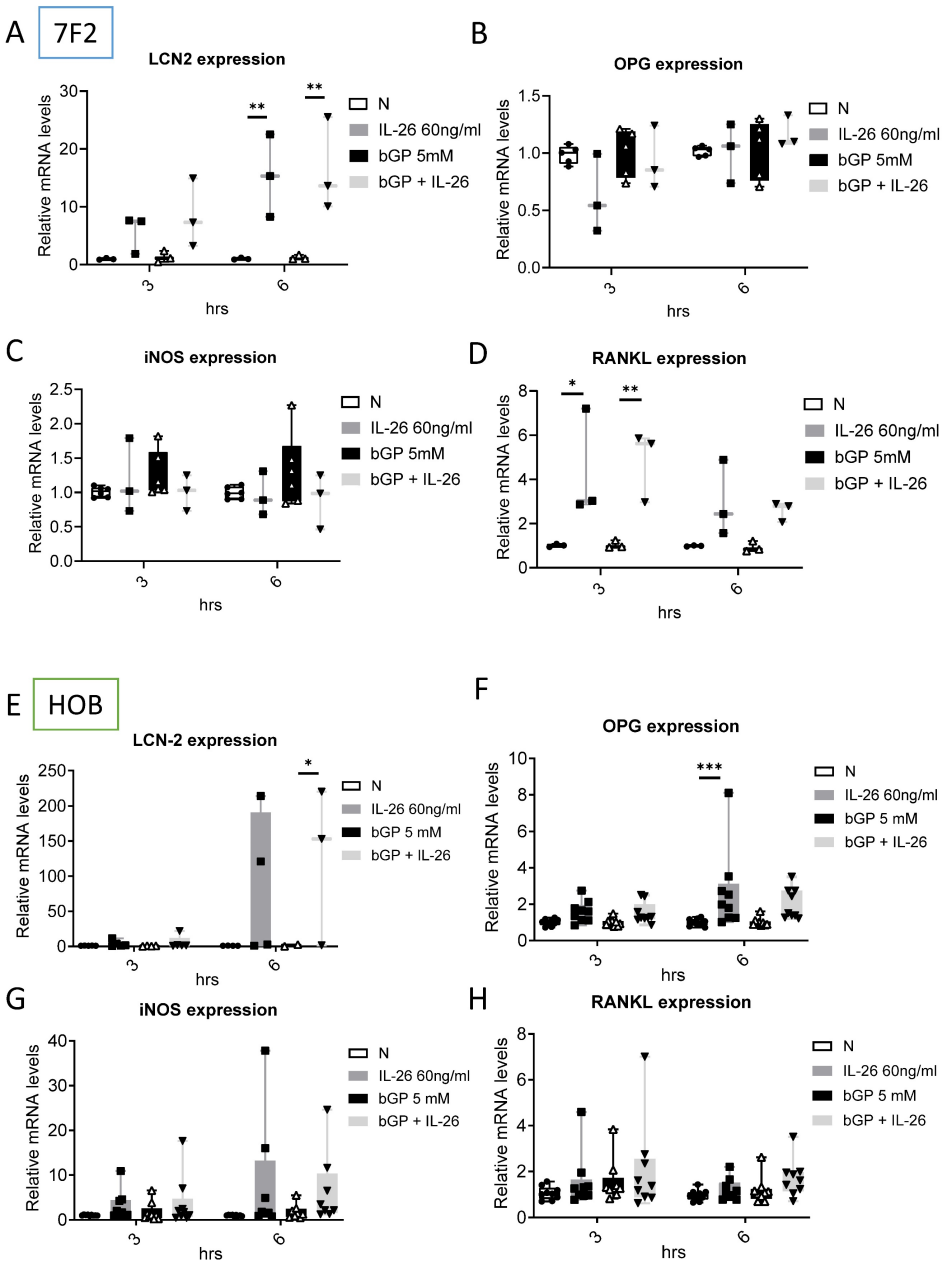
** Effects of IL-26 on the expressions of LCN2, OPG, iNOS and RANKL in 7F2 osteoblasts and HOB cells.** (A-D) 7F2 pre-osteoblasts and (E-H) HOB were differentiated using osteoblast differentiation medium in the presence or absence of IL-26 60 ng/ml. RNA extraction was performed at 3 and 6 hours after differentiation stimulation, following the previously described method. Real-time quantitative polymerase chain reaction (qPCR) was carried out using mouse and human-specific primers for LCN2, OPG, iNOS, and RANKL genes. The experiment was repeated at least three times, and the data represent the mean ± standard deviation. (*P<0.05, **P<0.01, ***P<0.001).

**Figure 5:**
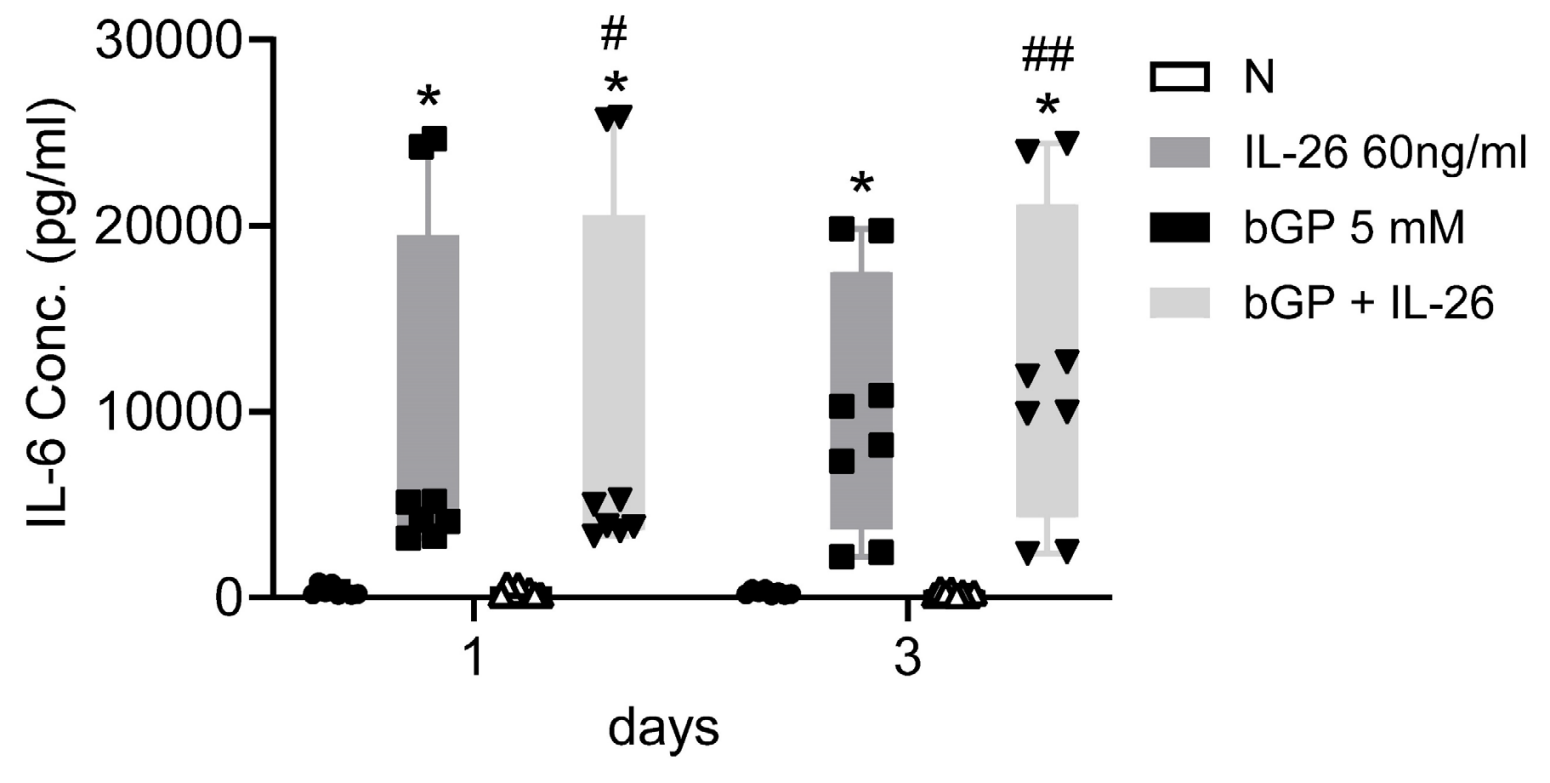
** IL-6 expression in HOB osteoblast differentiation during IL-26 stimulation.** HOB was differentiated using osteoblast differentiation medium in the presence or absence of IL-26 for 1 and 3 days. The condition medium was collected after treated IL-26 to HOB. (*P<0.05 compared to the N group; #P<0.05, ##P<0.01 compared to the bGP group).

To further elucidate the regulatory mechanisms governing communication in bone remodeling, we delved into the molecular dynamics of key factors. Lipocalin 2 (LCN2) was found to play a crucial role in various physiological conditions, exerting a negative regulatory effect on bone formation 22, 23. Inducible nitric oxide synthase (iNOS) activation, a common marker, is associated with localized osteolysis 24. OPG and Receptor Activator of Nuclear Factor Kappa-B Ligand (RANKL) are key factors in bone resorption, with the RANKL/OPG ratio determining bone mass and integrity 25. Our results showed a significant increase in LCN2 mRNA expression, with a 13-fold rise in 7F2 cells and a 123-fold rise in HOB cells six hours after IL-26 treatment (Fig. 4A, 4E), while RANKL expression increased only in 7F2 (Fig. 4D). Moreover, earlier research has indicated elevated concentrations of interleukin-6 (IL-6) in the synovial fluid of patients with end-stage OA 26. IL-6 stands out as one of the most prominently elevated cytokines implicated in the inflammatory response associated with OA 27. We further analyzed the condition medium after treated IL-26 to HOB. The result showed significantly upregulated of IL-6 expression, with an approximately 45-fold increase following IL-26 treatment (Fig. 5).

### Effects of IL-26 on nuclear translocation and activation of STAT1, COX-2, and NF-κB during osteoblast differentiation

**Figure 6:**
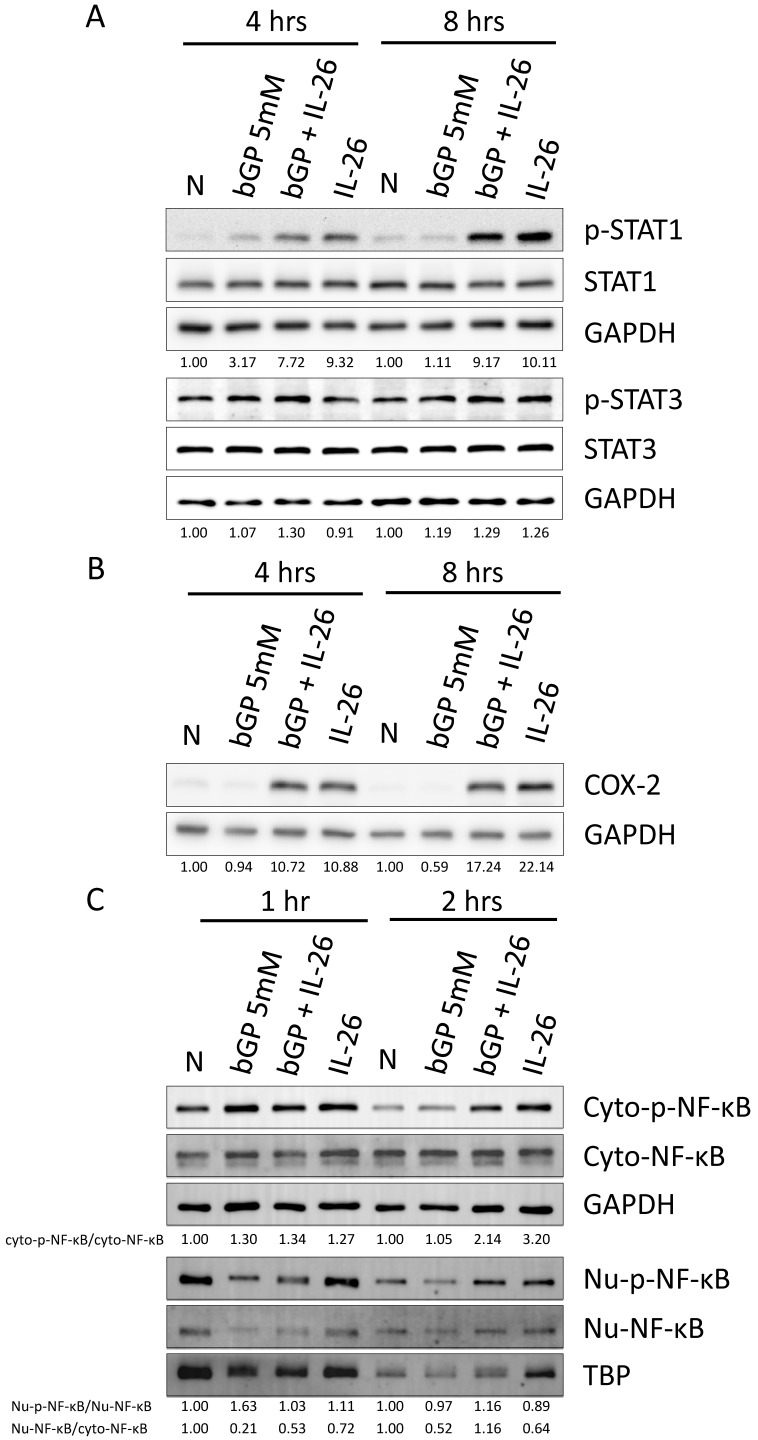
** Effects of IL-26 on signaling pathways during HOB osteoblast differentiation.** After 16 hours of serum-free culture, HOB was treated with osteoblast differentiation medium in the presence or absence of IL-26 for different hours. (A) Phosphorylated or non-phosphorylated STAT1, and STAT3, (B) COX-2 were analyzed by western blot. (C) Nuclear (Nu) and cytoplasmic (Cyto) extracts were analyzed by Western blot, and phosphorylated NF-κB proteins were detected using specific antibodies and compared with non-phosphorylated proteins. Electrophoresis was performed with the same amount of protein, TBP was used for the nucleus, and GAPDH was used as the control group for the cytoplasm.

In previous work, Hör *et al.* demonstrated that STAT1 and STAT3 play a critical role, being activated by IL-26 in colon cancer carcinoma 28. Therefore, we aimed to investigate whether IL-26 affects STAT1 and STAT3 in osteoblast cells during osteogenesis. Our results revealed that only STAT1 was activated and phosphorylated in HOB cells, with approximately a 2-fold increase at 4 hours and an 8-fold increase at 8 hours following IL-26 stimulation, compared to the bGP-alone group (Fig. 6A). Furthermore, NF-κB is a well-known upstream activator of COX-2, iNOS, LCN2, and RANKL 22, 23. The results showed that IL-26 stimulation significantly upregulated the expression of COX-2, resulting in an 11.4-fold increase at 4 hours and a 29.2-fold increase at 8 hours compared to the bGP-alone group (Fig. 6B). Subsequently, we analyzed whether NF-κB was activated and translocated to the nucleus after the administration of bGP and IL-26. Our results indicated a 2-fold increase in the phosphorylation and nuclear translocation of NF-κB during IL-26 stimulation of osteoblastogenesis (Fig. 6C).

## Discussion

In this research, we are trailblazing in discovering that IL-26 is directly expressed in inflammatory osteogenic cells, leading to the inhibition of osteoblastogenesis. Past research on IL-26 focused on its role in inflammation and immune responses as well as immune cells 29, 30. IL-26 expressed on macrophage and promoted macrophage from IL-9 expressing M1 subtype 31. Moreover, IL-26 plays a critical role in the first-line immune defense, such as neutralizing microbes, and is involved in respiratory, mucosal, and gut immunity 13, 32, 33. IL-26 also expressed on fibroblast and myofibroblast 34. All available evidence indicates that IL-26 functions as a responsive element to foreign pathogens, playing a role in the initiation of first-line immune inflammation and regulation. Here, we discovered that IL-26 can be expressed on osteogenic cells and regulate the differentiation of pathogenic osteoblasts.

Several pieces of evidence indicate that the dysregulation of osteoblasts plays a crucial role in the pathogenesis of osteoarthritis. Low OPG and high RANKL produced by osteoblasts lead to the activation of osteoclastogenesis in the early stage of the disease 35.

However, the RANKL/OPG ratio decreases in the moderate OA stage, promoting bone sclerosis. Our results showed that the murine 7F2 cell line exhibits higher RANKL expression, possibly due to osteoblast stimulation, similar to the early OA stage. Human osteoblasts were all derived from patients in the moderate or late stage, with high OPG sensitivity 36. Previous work report that osteoblast expresses the IL-20 receptor and transduce suppressive effect in osteoblastogenesis 37. The downstream molecular mechanism of IL-20R1, also a recognized shared receptor of IL-26, involves the activation of STAT3, inducing osteoblasts to produce RANKL and thereby promoting osteoclastogenesis 38. Our findings reveal that IL-26 enhances RANKL mRNA expression in murine 7F2 cells. Interestingly, IL-26 stimulates the underlying mechanisms more dramatically in activating STAT1 than STAT3 in suppression of osteoblast formation, distinguishing it from IL-20. Additionally, Kawane *et al.* state that RUNX2 is a crucial regulator of osteoblast differentiation, controlling precursor cell proliferation via fibroblast growth factor receptors Fgfr2 and Fgfr3 39. Heterozygous mutations in the RUNX2 gene were previously linked to Cleidocranial dysplasia 40. Mice lacking the RUNX2 gene exhibit delayed bone development and impaired intramembranous and endochondral ossification 41. Our results show that IL-26 partially inhibits RUNX2 expression in murine osteoblast. Although there is no significant difference in RUNX2 and RANKL gene expression in human HOB, this may be due to inherited induced gene tolerance and the fact that all patients were from the aging moderate or later OA stage in humans.

Heftdal *et al.* reported that IL-26 enhances osteoblast mineralization in spondyloarthritis 34, which contrasts with our findings. Interestingly, key differences include: first, cell source, our primary osteoblasts were derived from aging osteoarthritis patients, not commercial samples. Second, incubation time, we analyzed the full-time course of gene expression, not just the mineralization in terminal day; and third, absence of vitamin D supplementation during differentiation to study gene expression in both mice and humans. This decision is relevant, as BGLAP, also known as osteocalcin, is a protein produced by osteoblasts that plays a crucial role in bone metabolism, with its functions differing significantly between mice and humans. Vitamin D promotes BGLAP expression in human and rat osteoblasts but suppresses its expression in murine osteoblasts 42, 43. Moreover, mice exhibit differences in Bglap isoform expression, which is influenced by factors such as vitamin K and calcium, and they respond substantially to mechanical loading 44, 45. Human BGLAP has a more complex regulatory profile and a higher level of carboxylation 46, 47. While both species associate osteocalcin with energy metabolism, its effects are more pronounced in humans. These discrepancies underscore the importance of exercising caution when applying findings from mouse models to human health, particularly in studies of osteoporosis and metabolic diseases, as the role and regulation of osteocalcin may differ dramatically between species.

Prior research has demonstrated that LCN2, a participant in the innate immune system's germ-fighting mechanism, plays a regulatory role in maintaining bone balance 22, 48, 49. The overexpression of LCN2 through transient transfection in primary osteoblasts leads to the suppression of RUNX2 and ALP genes, along with hindering osteoblast differentiation 50. Additionally, LCN2 diminishes RANKL-induced IκBα phosphorylation, p65 nuclear import capacity, NF-κB, and NFATc1 transcriptional activity for osteoclast differentiation, suggesting potential implications of LCN2 in osteoclast suppressive function. 51. Our results show that IL-26 significantly increases LCN2 expression, accompanied by the inhibition of osteoblast formation. Combined with previous findings, IL-26 has been shown to suppress osteoclastogenesis. IL-26 may play an essential role in reducing the bone turnover rate in inflammatory bone homeostasis, and the underlying molecular mechanisms may be transduced by the iNOS, COX2, STAT1, and NF-κB pathways. Despite the absence of IL-26 protein in mice, there has been limited cross-species research on osteoblast differentiation. Regarding the regulation of osteoblast differentiation, the human primary cultured HOB cells and the mouse 7F2 cell line show some differences. Nevertheless, we found that IL-26 plays a role in controlling osteoblast activity and maintaining bone homeostasis.

In conclusion, we have presented new evidence indicating that IL-26 is directly expressed by osteogenic cells, autonomously suppresses osteoblastogenesis and regulates osteoblast-related genes. Our findings support the notion that IL-26 induces osteoblast suppression through signaling pathways involving STAT1, COX2, and NF-κB. Furthermore, IL-26-stimulated osteoblasts enhance the emergency immune response by inducing the expression of inducible iNOS and the proinflammatory cytokine IL-6, thereby regulating the immune system. In summary, our findings decipher the crucial role of IL-26 in the regulation of osteoblast cells and bone homeostasis, suggesting that IL-26 could be a potential therapeutic target for inflammatory bone diseases such as OA.

## Figures and Tables

**Table 1:** Primer used for quantitative real-time PCR.

Genes	Nucleotide sequences	Reference
m/h-GAPDH	Forward GTGAGGCCGGTGCTGAGTATGT	
	Reverse ACAGTCTTCTGGGTGGCAGTGAT	52
m-osteocalcin (BGLAP)	Forward TGACAAAGCCTTCATGTCCAAG	53
	Reverse ACAGGGAGGATCAAGTCCC	
m-RUNX2	Forward GGAACCAAGAAGGCACAGAC	54
	Reverse TGGATGGGGATGTCATCTGG	
m-osteopontin (Spp1)	Forward CTGGCTGAATTCTGAGGGACT	55
	Reverse TTCTGTGGCGCAAGGAGATT	
m-ALP	Forward CCAACTCTTTTGTGCCAGAGA	56
	Reverse GGCTACATTGGTGTTGAGCTTTT	
m-LCN2	Forward CCAGTTCGCCATGGTATTTT	57
	Reverse CACACTCACCACCCATTCAG	
m-OPG	Forward AAAGCACCCTGTAGAAAACA	58
	Reverse CCGTTTTATCCTCTCTACACTC	
m-iNOS	Forward CTTTGCCACGGACGAGAC	59
	Reverse TCATTGTACTCTGAGGGCTGA C	
m-RANKL	Forward AGCCATTTGCACACCTCAC	60
	Reverse CGTGGTACCAAGAGGACAGAGT	
h-osteocalcin (BGLAP)	Forward CACCGAGACACCATGAGAGC	61
	Reverse CTGCTTGGACACAAAGGCTGC	
h-RUNX2	Forward GCGGTGCAAACTTTCTCCAG	62
	Reverse TGCTTGCAGCCTTAAATGACTC	
h-osteopontin (Spp1)	Forward AACGCCGACCAAGGAAAAC	
	Reverse AGCTTCTGGGGACAATGCC	
h-ALP	Forward GGGTCAGCTCCACCACAA	63
	Reverse GGCATTGGTGTTGTACGTCTT	
h-LCN2	Forward TTCCCTGTCCCAATCGACCA	64
	Reverse TTTAGCAGACAAGGTGGGGC	
h-OPG	Forward GAAGGGCGCTACCTTGAGAT	65
	Reverse GCAAACTGTATTTCGCTCTGG	
h-iNOS	Forward TCTCAAGGCACAGGTCTCTTC	66
	Reverse GTTCTTCACTGTGGGGCTTG	
h-RANKL	Forward GTTCGTGGCCCTCCTGG	
	Reverse GATCCATCTGCGCTCTGAAA	

## References

[1] Kapoor M, Martel-Pelletier J, Lajeunesse D, Pelletier JP, Fahmi H (2011). Role of proinflammatory cytokines in the pathophysiology of osteoarthritis. Nat Rev Rheumatol.

[2] Kragstrup TW, Sohn DH, Lepus CM, Onuma K, Wang Q, Robinson WH (2019). Fibroblast-like synovial cell production of extra domain A fibronectin associates with inflammation in osteoarthritis. BMC rheumatology.

[3] Siddiqui JA, Le Henaff C, Johnson J, He Z, Rifkin DB, Partridge NC (2021). Osteoblastic monocyte chemoattractant protein-1 (MCP-1) mediation of parathyroid hormone's anabolic actions in bone implicates TGF-β signaling. Bone.

[4] Chen G, Deng C, Li YP (2012). TGF-β and BMP signaling in osteoblast differentiation and bone formation. Int J Biol Sci.

[5] Le Henaff C, Mansouri R, Modrowski D, Zarka M, Geoffroy V, Marty C (2015). Increased NF-κB Activity and Decreased Wnt/β-Catenin Signaling Mediate Reduced Osteoblast Differentiation and Function in ΔF508 Cystic Fibrosis Transmembrane Conductance Regulator (CFTR) Mice. J Biol Chem.

[6] Biskobing DM, Fan X, Rubin J (1995). Characterization of MCSF-induced proliferation and subsequent osteoclast formation in murine marrow culture. J Bone Miner Res.

[7] Simonet WS, Lacey DL, Dunstan CR, Kelley M, Chang MS, Lüthy R (1997). Osteoprotegerin: a novel secreted protein involved in the regulation of bone density. Cell.

[8] Glass DA 2nd, Bialek P, Ahn JD, Starbuck M, Patel MS, Clevers H (2005). Canonical Wnt signaling in differentiated osteoblasts controls osteoclast differentiation. Dev Cell.

[9] Dapunt U, Giese T, Stegmaier S, Moghaddam A, Hänsch GM (2016). The osteoblast as an inflammatory cell: production of cytokines in response to bacteria and components of bacterial biofilms. BMC Musculoskelet Disord.

[10] Sheikh F, Baurin VV, Lewis-Antes A, Shah NK, Smirnov SV, Anantha S (2004). Cutting edge: IL-26 signals through a novel receptor complex composed of IL-20 receptor 1 and IL-10 receptor 2. Journal of immunology.

[11] Itoh T, Hatano R, Komiya E, Otsuka H, Narita Y, Aune TM (2019). Biological Effects of IL-26 on T Cell-Mediated Skin Inflammation, Including Psoriasis. J Invest Dermatol.

[12] Donnelly RP, Sheikh F, Dickensheets H, Savan R, Young HA, Walter MR (2010). Interleukin-26: an IL-10-related cytokine produced by Th17 cells. Cytokine Growth Factor Rev.

[13] Che KF, Tengvall S, Levanen B, Silverpil E, Smith ME, Awad M (2014). Interleukin-26 in antibacterial host defense of human lungs. Effects on neutrophil mobilization. American journal of respiratory and critical care medicine.

[14] Corvaisier M, Delneste Y, Jeanvoine H, Preisser L, Blanchard S, Garo E (2012). IL-26 is overexpressed in rheumatoid arthritis and induces proinflammatory cytokine production and Th17 cell generation. PLoS Biol.

[15] Peng YJ, Wang CY, Lin YH, Lin GJ, Huang SH, Shyu JF (2016). Interleukin 26 suppresses receptor activator of nuclear factor kappaB ligand induced osteoclastogenesis via down-regulation of nuclear factor of activated T-cells, cytoplasmic 1 and nuclear factor kappaB activity. Rheumatology (Oxford).

[16] Lin YH, Wang YH, Peng YJ, Liu FC, Lin GJ, Huang SH (2020). Interleukin 26 Skews Macrophage Polarization Towards M1 Phenotype by Activating cJUN and the NF-kappaB Pathway. Cells.

[17] Chang SF, Yeh CC, Chen PJ, Chang HI (2018). The Impact of Lipid Types and Liposomal Formulations on Osteoblast Adiposity and Mineralization. Molecules (Basel, Switzerland).

[18] Qiu W, Hu Y, Andersen TE, Jafari A, Li N, Chen W (2010). Tumor necrosis factor receptor superfamily member 19 (TNFRSF19) regulates differentiation fate of human mesenchymal (stromal) stem cells through canonical Wnt signaling and C/EBP. J Biol Chem.

[19] Neve A, Corrado A, Cantatore FP (2013). Osteocalcin: skeletal and extra-skeletal effects. J Cell Physiol.

[20] Singh A, Gill G, Kaur H, Amhmed M, Jakhu H (2018). Role of osteopontin in bone remodeling and orthodontic tooth movement: a review. Prog Orthod.

[21] Kruger TE, Miller AH, Godwin AK, Wang J (2014). Bone sialoprotein and osteopontin in bone metastasis of osteotropic cancers. Crit Rev Oncol Hematol.

[22] Li C, Chan YR (2011). Lipocalin 2 regulation and its complex role in inflammation and cancer. Cytokine.

[23] Veeriah V, Zanniti A, Paone R, Chatterjee S, Rucci N, Teti A (2016). Interleukin-1β, lipocalin 2 and nitric oxide synthase 2 are mechano-responsive mediators of mouse and human endothelial cell-osteoblast crosstalk. Scientific reports.

[24] van't Hof RJ, Armour KJ, Smith LM, Armour KE, Wei XQ, Liew FY (2000). Requirement of the inducible nitric oxide synthase pathway for IL-1-induced osteoclastic bone resorption. Proc Natl Acad Sci U S A.

[25] Boyce BF, Xing L (2007). Biology of RANK, RANKL, and osteoprotegerin. Arthritis Res Ther.

[26] Beekhuizen M, Gierman LM, van Spil WE, Van Osch GJ, Huizinga TW, Saris DB (2013). An explorative study comparing levels of soluble mediators in control and osteoarthritic synovial fluid. Osteoarthritis and cartilage.

[27] Akeson G, Malemud CJ (2017). A Role for Soluble IL-6 Receptor in Osteoarthritis. Journal of functional morphology and kinesiology.

[28] Hör S, Pirzer H, Dumoutier L, Bauer F, Wittmann S, Sticht H (2004). The T-cell lymphokine interleukin-26 targets epithelial cells through the interleukin-20 receptor 1 and interleukin-10 receptor 2 chains. J Biol Chem.

[29] Miot C, Beaumont E, Duluc D, Le Guillou-Guillemette H, Preisser L, Garo E (2015). IL-26 is overexpressed in chronically HCV-infected patients and enhances TRAIL-mediated cytotoxicity and interferon production by human NK cells. Gut.

[30] Manel N, Unutmaz D, Littman DR (2008). The differentiation of human T(H)-17 cells requires transforming growth factor-beta and induction of the nuclear receptor RORgammat. Nat Immunol.

[31] Wang YH, Peng YJ, Liu FC, Lin GJ, Huang SH, Sytwu HK (2023). Interleukin 26 Induces Macrophage IL-9 Expression in Rheumatoid Arthritis. Int J Mol Sci.

[32] Cella M, Fuchs A, Vermi W, Facchetti F, Otero K, Lennerz JK (2009). A human natural killer cell subset provides an innate source of IL-22 for mucosal immunity. Nature.

[33] Song D, Lai L, Lu J, Tong J, Ran Z (2022). Interleukin-26 Expression in Inflammatory Bowel Disease and Its Immunoregulatory Effects on Macrophages. Front Med (Lausanne).

[34] Heftdal LD, Andersen T, Jaehger D, Woetmann A, Ostgard R, Kenngott EE (2017). Synovial cell production of IL-26 induces bone mineralization in spondyloarthritis. J Mol Med (Berl).

[35] Kwan Tat S, Pelletier JP, Lajeunesse D, Fahmi H, Lavigne M, Martel-Pelletier J (2008). The differential expression of osteoprotegerin (OPG) and receptor activator of nuclear factor kappaB ligand (RANKL) in human osteoarthritic subchondral bone osteoblasts is an indicator of the metabolic state of these disease cells. Clin Exp Rheumatol.

[36] Muratovic D, Atkins GJ, Findlay DM (2024). Is RANKL a potential molecular target in osteoarthritis?. Osteoarthritis Cartilage.

[37] Hsu YH, Chiu YS, Chen WY, Huang KY, Jou IM, Wu PT (2016). Anti-IL-20 monoclonal antibody promotes bone fracture healing through regulating IL-20-mediated osteoblastogenesis. Sci Rep.

[38] Hsu YH, Chen WY, Chan CH, Wu CH, Sun ZJ, Chang MS (2011). Anti-IL-20 monoclonal antibody inhibits the differentiation of osteoclasts and protects against osteoporotic bone loss. J Exp Med.

[39] Kawane T, Qin X, Jiang Q, Miyazaki T, Komori H, Yoshida CA (2018). Runx2 is required for the proliferation of osteoblast progenitors and induces proliferation by regulating Fgfr2 and Fgfr3. Scientific reports.

[40] Zhang YW, Yasui N, Kakazu N, Abe T, Takada K, Imai S (2000). PEBP2alphaA/CBFA1 mutations in Japanese cleidocranial dysplasia patients. Gene.

[41] Komori T, Yagi H, Nomura S, Yamaguchi A, Sasaki K, Deguchi K (1997). Targeted disruption of Cbfa1 results in a complete lack of bone formation owing to maturational arrest of osteoblasts. Cell.

[42] Yoshizawa T, Handa Y, Uematsu Y, Takeda S, Sekine K, Yoshihara Y (1997). Mice lacking the vitamin D receptor exhibit impaired bone formation, uterine hypoplasia and growth retardation after weaning. Nat Genet.

[43] Zhang R, Ducy P, Karsenty G (1997). 1,25-dihydroxyvitamin D3 inhibits Osteocalcin expression in mouse through an indirect mechanism. J Biol Chem.

[44] Gundberg CM, Lian JB, Booth SL (2012). Vitamin K-dependent carboxylation of osteocalcin: friend or foe?. Adv Nutr.

[45] Komori T (2020). Functions of Osteocalcin in Bone, Pancreas, Testis, and Muscle. Int J Mol Sci.

[46] Zoch ML, Clemens TL, Riddle RC (2016). New insights into the biology of osteocalcin. Bone.

[47] Wang JS, Mazur CM, Wein MN (2021). Sclerostin and Osteocalcin: Candidate Bone-Produced Hormones. Front Endocrinol (Lausanne).

[48] Goetz DH, Holmes MA, Borregaard N, Bluhm ME, Raymond KN, Strong RK (2002). The neutrophil lipocalin NGAL is a bacteriostatic agent that interferes with siderophore-mediated iron acquisition. Molecular cell.

[49] Xu S, Venge P (2000). Lipocalins as biochemical markers of disease. Biochim Biophys Acta.

[50] Rucci N, Capulli M, Piperni SG, Cappariello A, Lau P, Frings-Meuthen P (2015). Lipocalin 2: a new mechanoresponding gene regulating bone homeostasis. J Bone Miner Res.

[51] Kim HJ, Yoon HJ, Yoon KA, Gwon MR, Jin Seong S, Suk K (2015). Lipocalin-2 inhibits osteoclast formation by suppressing the proliferation and differentiation of osteoclast lineage cells. Experimental cell research.

[52] Porter KA, Kelley LN, Nekorchuk MD, Jones JH, Hahn AB, de Noronha CM (2010). CIITA enhances HIV-1 attachment to CD4+ T cells leading to enhanced infection and cell depletion. J Immunol.

[53] Yang Y, Chen D, Li Y, Zou J, Han R, Li H (2022). Effect of Puerarin on Osteogenic Differentiation in vitro and on New Bone Formation in vivo. Drug Des Devel Ther.

[54] Schmid R, Bosserhoff AK (2014). Redundancy in regulation of chondrogenesis in MIA/CD-RAP-deficient mice. Mech Dev.

[55] Linder M, Hecking M, Glitzner E, Zwerina K, Holcmann M, Bakiri L (2018). EGFR controls bone development by negatively regulating mTOR-signaling during osteoblast differentiation. Cell Death Differ.

[56] Jun JH, Yoon WJ, Seo SB, Woo KM, Kim GS, Ryoo HM (2010). BMP2-activated Erk/MAP kinase stabilizes Runx2 by increasing p300 levels and histone acetyltransferase activity. J Biol Chem.

[57] Coban C, Ishii KJ, Uematsu S, Arisue N, Sato S, Yamamoto M (2007). Pathological role of Toll-like receptor signaling in cerebral malaria. Int Immunol.

[58] Perez M, Migliaccio S, Taranta A, Festuccia C, Orru L, Brama M (2001). Melanoma cells stimulate osteoclastogenesis, c-Src expression and osteoblast cytokines. Eur J Cancer.

[59] Degrandi D, Konermann C, Beuter-Gunia C, Kresse A, Wurthner J, Kurig S (2007). Extensive characterization of IFN-induced GTPases mGBP1 to mGBP10 involved in host defense. J Immunol.

[60] Swarnkar G, Sharan K, Siddiqui JA, Mishra JS, Khan K, Khan MP (2012). A naturally occurring naringenin derivative exerts potent bone anabolic effects by mimicking oestrogen action on osteoblasts. Br J Pharmacol.

[61] Tao SC, Gao YS, Zhu HY, Yin JH, Chen YX, Zhang YL (2016). Decreased extracellular pH inhibits osteogenesis through proton-sensing GPR4-mediated suppression of yes-associated protein. Sci Rep.

[62] Zhang Y, Weng S, Yin J, Ding H, Zhang C, Gao Y (2017). Vitamin K2 promotes mesenchymal stem cell differentiation by inhibiting miR-133a expression. Mol Med Rep.

[63] Spreafico A, Chellini F, Frediani B, Bernardini G, Niccolini S, Serchi T (2009). Biochemical investigation of the effects of human platelet releasates on human articular chondrocytes. J Cell Biochem.

[64] Overcast GR, Meibers HE, Eshleman EM, Saha I, Waggoner L, Patel KN (2023). IEC-intrinsic IL-1R signaling holds dual roles in regulating intestinal homeostasis and inflammation. J Exp Med.

[65] Rauner M, Goettsch C, Stein N, Thiele S, Bornhaeuser M, De Bosscher K (2011). Dissociation of osteogenic and immunological effects by the selective glucocorticoid receptor agonist, compound A, in human bone marrow stromal cells. Endocrinology.

[66] Balaz M, Becker AS, Balazova L, Straub L, Muller J, Gashi G (2019). Inhibition of Mevalonate Pathway Prevents Adipocyte Browning in Mice and Men by Affecting Protein Prenylation. Cell Metab.

